# Integrated Phenotypic-Genotypic Analysis of Candidate Probiotic *Weissella Cibaria* Strains Isolated from Dairy Cows in Kuwait

**DOI:** 10.1007/s12602-020-09715-x

**Published:** 2020-10-21

**Authors:** Vania Patrone, Tahani Al-Surrayai, Francesco Romaniello, Alessandra Fontana, Giovanni Milani, Valeria Sagheddu, Edoardo Puglisi, Maria Luisa Callegari, Hamad Al-Mansour, Mohamed Waheed Kishk, Lorenzo Morelli

**Affiliations:** 1 Department for Sustainable Food Process (DiSTAS), Università Cattolica del Sacro Cuore, via E. Parmense 84, 29122 Piacenza, Italy; 2 Kuwait Institute for Scientific Research, Shuwaikh area, Kuwait; 3 Biotechnological Research Centre, Università Cattolica del Sacro Cuore, via Milano 24, 26100 Cremona, Italy; 4 AAT - Advanced Analytical Technologies Srl, Via P. Majavacca 12, 29107 Fiorenzuola d’Arda (Piacenza), Italy

**Keywords:** *Weissella*, Probiotics, Cattle, Genomics, Antagonistic activity, Intestinal disease

## Abstract

Probiotics represent a possible strategy for controlling intestinal infections in livestock. Members of the *Weissella* genus are increasingly being studied for health-related applications in animals and humans. Here we investigated the functional properties of two *Weissella cibaria* strains isolated from cows reared in Kuwait breeding facilities by combining phenotypic with genomic analyses. *W. cibaria* SP7 and SP19 exhibited good growth in vitro under acidic conditions and in the presence of bile salts compared to the reference probiotic *Lacticaseibacillus* (formerly *Lactobacillus*) *rhamnosus* GG. Both strains were able to adhere to Caco-2 and HT-29 cell lines, as well as to mucin. The cell-free supernatants of the two isolates exhibited inhibitory activity towards *Escherichia coli* ATCC 25,922 and *Salmonella enterica* UC3605, which was ultimately due to the low pH of supernatants. *W. cibaria* SP19 showed a co-aggregation ability similar to that of *L. rhamnosus* GG when incubated with *S. enterica*. Whole genome sequencing and analysis revealed that both strains harbored several genes involved in carbohydrate metabolism and general stress responses, indicating bacterial adaptation to the gastrointestinal environment. We also detected genes involved in the adhesion to host epithelial cells or extracellular matrix. No evidence of acquired antibiotic resistance or hemolytic activity was found in either strain. These findings shed light on the potential of *W. cibaria* for probiotic use in livestock and on the mechanisms underlying host-microbe interaction in the gut. *W. cibaria`* strain SP19 exhibited the best combination of in vitro probiotic properties and genetic markers, and is a promising candidate for further investigation.

## Introduction

Over the last decade, there has been a considerable increase in the use of probiotics in animal feeding, with the aim of improving animal performance, digestion, productivity, and resistance to infectious diseases [1, 2]. Infection control is a serious problem for cattle facilities in Kuwait, where dairy farms are managed in an intensive system due to the extremely arid and harsh environment. The type of housing puts stress on animals and, as a consequence, dairy farmers are faced with a high calf mortality rate that is mainly attributed to enterotoxaemia, diarrhea, and pneumonia caused by *Pasteurella*.

Among lactic acid bacteria, the probiotic potential of *Weissella* spp. has only recently been investigated in humans as well as in animals. Several studies have reported *Weissella* strains, mostly belonging to the species *Weissella cibaria*, *Weissella confusa* and *Weissella paramesenteroides*, to possess some functional properties relevant to their use as probiotics [3–6]. Notably, *W. cibaria* JW15 was found to possess anti-oxidant and anti-inflammatory properties in vitro [7, 8], and to exert immune-modulatory effects in mice [9] and in human subjects [10]. *W. cibaria* WIKIM28 had also important immune effects in mice, suppressing allergic Th2 responses and inducing Treg responses in atopic dermatitis [11]. In addition, *W. cibaria* has been suggested as oral care probiotics: strain CMU has shown significant antimicrobial activity against oral pathogens [12] and a beneficial impact on halitosis and periodontitis in animals and humans [13–15].

The development of efficient probiotics relies on a proper selection of bacterial strains to secure the desired benefit in the target host. This implies the absence of pathogenic traits, the capacity to survive during passage and colonize the gastrointestinal tract, as well as to produce antimicrobial substances against pathogens. In the light of recent findings showing that colonization of the intestine may be highly dependent on the host genotype and gut microbiome [16], it is reasonable to assume that better performing strains may be identified if they are specifically selected for the target population. *Weissella* spp. seem to be well adapted to cattle, in that they have been often identified in cow feces [17, 18], vagina [19], skin [20], and milk [21, 22]. Recently, *Weissella* spp. were isolated from hosts that have a high capacity for adaptation to survive in arid and desert areas characterized by poor nutrients, high temperatures, and desiccation, such as camels [23]. The authors of the study hypothesized that organisms living in arid lands may select specific gut commensals with special metabolic characteristics contributing to the adaptation of their hosts to harsh environmental conditions.

In this study we evaluated the probiotic potential of two *W. cibaria* strains selected from among a range of lactic acid bacteria isolated from dairy cows in Kuwait; we sought to make a preliminary assessment from the perspective of developing a tailored treatment strategy to support calf intestinal health. We used conventional in vitro assays to determine *W. cibaria* resistance to acid and bile stress, adhesion ability, and antimicrobial activity against pathogens. Whole genome sequencing and sequence analysis was used as a complementary approach to deeply investigate the safety and probiotic potential of these promising candidates, as well as to gain insight into the genome evolutionary strategies adopted by these two strains to adapt to the gastrointestinal tract and interact with the host.

## Material and Methods

### Sample Collection, Strain Isolation, and Identification

Sampling was carried out on two dairy farms (Al-Adwani and Al-Dhbabi) located in the Kabd area of Kuwait. A sampling schedule was prepared to cover the four different seasons: winter, spring, summer, and autumn. Fecal samples were collected from healthy adult cows and calves, preserved in an ice box, and transported to the laboratory for analysis. Milk samples were collected in sterile glass bottles, refrigerated, and transported to the laboratory where they were analyzed immediately. A total of 26 samples were collected. Fecal samples were obtained with informed written consent from the owners of the animals. All efforts were made to minimize animal discomfort according to high standards of veterinary care. No experimentation was done on the animals.

Each 10 g sample was mixed with 90 mL of sterile saline solution (0.85% w/v NaCl) and homogenized using a Stomacher machine (400 Circulator, International PBI, Milan, Italy) at 260 rpm for 1.5 min. One milliliter aliquots of the homogenate were serially diluted in 9 mL of saline and used to isolate strains on MRS agar medium (Merck, Darmstad, Germany) incubated at 37 °C for 48 h in anaerobic jars. No antifungal agents were added in order to avoid any possible interference with LAB growth. Different bacterial colonies were selected on the basis of morphological characteristics (small greyish white colonies) and subjected to three further purification steps under the same anaerobic conditions. Gram-positive and catalase-negative isolates were retained as putative LAB and their identities were confirmed by 16S rRNA amplicon sequencing. Two *W. cibaria* strains, identified as SP7 and SP19, were selected for further analysis from among all LAB cultures recovered from cow feces. The known probiotic *Lacticaseibacillus* (formerly *Lactobacillus*) *rhamnosus* GG was included as a control strain.

### In Vitro Phenotypic Assays

#### pH and Bile Salt Tolerance

The acid tolerance of *W. cibaria* strains and *L. rhamnosus* GG was determined by exposing bacterial cells to low pH. The strains were grown in MRS broth at 37 °C overnight and centrifuged at 3000 rpm for 10 min at 4 °C; the pellets were washed in sterile phosphate-buffered saline (PBS, pH 7) and then suspended in PBS [optical density at 600 nm (OD_600nm)_ = 0.8]. An aliquot (0.15 mL) of each washed cell suspension was added to 1.485 mL of PBS at pH 2, 3, 4, and 7 (control). The mixture was then vortexed at maximum speed for 10 s and incubated at 37 °C. An aliquot of 0.1 mL was removed after 1, 2, and 4 h, serially diluted in 0.85% sterile saline, and plated on MRS agar to determine the total viable count [24].

Bile salt tolerance was evaluated by inoculating 1 mL of bacterial suspension (OD_600nm_ = 0.8) in 9 mL of MRS broth supplemented with 0 (control), 0.5, and 2% (w/v) Ox-bile dehydrated purified (Merck KGaA, Germany). After thorough mixing, the test tubes were incubated at 37 °C. One milliliter of culture was taken from each tube immediately (0 h) and after 2 h incubation, and a series of tenfold dilutions were prepared. Dilutions were plated on MRS agar and incubated at 37 °C for 48 h [25]. Experiments were performed in triplicate.

#### Aggregation and Co-Aggregation Tests

Auto-aggregation assays were performed according to Kos et al. [26]. Pellets of bacterial cultures grown overnight in MRS broth were collected by centrifugation at 5000 rpm for 15 min, washed twice in PBS (pH 7), and re-suspended in PBS after adjusting the OD_600nm_ to 1 (approximately 10^7^ CFU mL^−1^). Cell suspensions (4 mL) were mixed by vortexing for 10 s and auto-aggregation measured immediately (t = 0) and after 1, 2, 3, and 4 h of incubation at 37 °C. For every hour interval, 100 µL from the upper suspension was transferred to another tube with 3.9 mL of PBS and the absorbance (A) measured at 600 nm. The auto-aggregation was expressed as a percentage determined by 1-(A_t_/A_0_) × 100, where A_t_ represents the absorbance at time (t) = 1, 2, 3, or 4 h and A_0_ the absorbance at t = 0.

For co-aggregation tests, bacterial strains were grown anaerobically at 37 °C for 18 h in MRS broth. *Salmonella enterica* UC3605 from the Università Cattolica del Sacro Cuore Bacteria Culture Collection and *Escherichia coli* ATCC 25,922 were used as test pathogens. Suspensions of *W. cibaria* strains and test pathogens were adjusted to an OD_600nm_ of 0.5. Two milliliters of each LAB strain suspension were mixed with the same volume of test pathogen, blended on a vortex mixer for 10 s, and incubated at 37 °C. Control tubes contained 4.0 mL of the bacterial suspension of each strain alone. The OD_600nm_ of the resulting suspensions was measured immediately (t = 0) and after 1, 2, 3, and 4 h by transferring 100 µL from the upper suspension to another tube with 3.9 mL of PBS as described previously. Co-aggregation was calculated as a percentage according to the equation described by Handley et al. [27]. Experiments were performed in triplicate.

#### Adhesion to Cell Lines and Porcine Gastric Mucin

The mucin and eukaryotic cell adhesion assays were carried out for all strains using porcine gastric mucin, type II (Sigma-Aldrich, Germany) and Caco-2 and HT-29 cells obtained from European Collection of Authenticated Cell Cultures (ECACC). Culturing, maintenance, and adhesion tests were performed as described previously [28, 29] with minor modifications. Strains were inoculated in MRS broth and incubated for 24 h at 37 °C under anaerobic conditions. Broth cultures were centrifuged and pellets washed twice with Hank's balanced salt solution (HBSS). Washed cells were resuspended and diluted to 1.5 × 10^8^ CFU mL^−1^. The bacterial suspensions were further diluted 1:10 in high glucose Dulbecco's Modified Eagle Medium (DMEM), and 125 µL of these dilutions were used to inoculate the wells seeded with Caco-2 or HT-29 cells, or mucin-coated and the other control wells in order to obtain a multiplicity of infection (MOI) of 5:1.

Cell lines or mucin plus probiotic strains were incubated for 1 h at 37 °C with 5% of CO_2_. After incubation, the volume in the control wells was harvested and serially diluted and plated on MRS agar according to Rapporti Istisan 08/36 [30]. The medium was removed from the seeded wells and the Caco-2 or HT-29 cell monolayers and mucin coating washed three times with 1 mL of HBSS for 5 min. The wells seeded with eukaryotic cells were inoculated with 100 µL trypsin, and the mucin-coated wells with 100 µL of a 0.5% solution of Triton-X and incubated for 5 min at 37 °C to break the cell monolayers and mucus coating, respectively. The homogenates composed of cell lines or mucin and probiotics were recovered with 900 µL of Maximum Recovery Diluent (MRD, BD Difco, England), serially diluted, and plated onto MRS agar. Count plates were incubated for 72 h at 37 °C under anaerobic conditions.

The adhesion percentages were obtained by referring to the following formula: *P* = (µ/M) × 100, where *P* represents the percentage of tested strains adhering to the human cells or mucin, µ represents the vital count of analyzed strains bonded to intestinal cell lines or mucin expressed as a logarithmic value, and M represents the vital count of analyzed strains transformed as a logarithmic value in the wells without human cells or mucin coating.

#### Antagonistic Activity Against Pathogens

The method in Choi et al. [31] was used with minor modifications. After 24 h at 37 °C, *W. cibaria* MRS broth cultures were centrifuged at 4000 rpm for 15 min and filtered using a 0.22 µm filter (Jet Biofil, Guangzhou, China) to obtain the cell-free supernatant (CFS). *S. enterica* UC3605 and *E. coli* ATCC 25,922 were incubated in Luria Broth for 24 h, diluted in 2X Luria Broth to an OD_600nm_ of 0.06, corresponding to McFarland standard 0.5, and then 50 μL was added to each well of a microtiter plate. The same volume of CFS from *W. cibaria* strains was added to each well and incubated at 37 °C for 24 h; filtered CFS was tested either after neutralization to pH 6.5 or without pH neutralization. In the control trials, sterile and neutralized MRS broth was used instead of the supernatant. The growth of pathogens was determined by measuring the OD_600nm_ after 24 h. Tests were conducted in duplicate.

#### Carbohydrate Fermentation Patterns and Exopolysaccharide (EPS) Production

Carbohydrate utilization profiles of *W. cibaria* SP7 and SP19 were assessed using API 50 CH strips (bioMérieux, Marcy l’Etoile, France) in API 50 CHL Lactobacilli broth as indicated by the manufacturer. The inoculated strips were incubated at 37 °C in anaerobiosis and the reactions observed after 48 h.

The *W. cibaria* strains were screened for their ability to produce EPS using modified MRS (mod-MRS) agar medium (Proteose Peptone No. 3 10 gL^−1^, Beef extract 10 gL^−1^, Yeast extract 5 gL^−1^, Polysorbate 80 1 gL^−1^, Ammonium Citrate 2 gL^−1^, Sodium Acetate 5 gL^−1^, Magnesium Sulfate 0.1 gL^−1^, Manganese Sulfate 0.05 gL^−1^, Dipotassium Phosphate 2 gL^−1^, Agar 15 gL^−1^), testing individual sugars that had a positive fermentation output in the carbohydrate fermentation pattern assessment. Mod-MRS agar plates containing 20 g L^−1^ of glucose, fructose, D-maltose, sucrose, galactose, L-arabinose, D-ribose, D-xylose, D-mannose, D-cellobiose, or gentiobiose (Sigma-Aldrich, Germany) as the sole source of carbohydrate were streaked with the *W. cibaria* isolates and incubated at 37 °C for 2 to 3 days. The detection of EPS production was facilitated by the addition of Ruthenium Red [0.08% (w/v); Sigma-Aldrich, Germany] [32]. Tests were conducted in duplicate.

#### Safety Evaluation of *W. cibaria* Strains

The antibiotic susceptibility profiles of the two *W. cibaria* strains were determined by broth microdilution according to the ISO 10,932/IDF 233 standard [33] and EFSA guidelines [34] with VetMIC Lact-1 and Lact-2 plates (SVA National Veterinary Institute, Uppsala, Sweden). Briefly, bacterial colonies were suspended in 5 mL of sterile saline solution (0.9% NaCl) to obtain a density corresponding to McFarland standard 1. Suspensions were diluted 1:1000 in LAB susceptibility test medium (LSM); 100 μL of this dilution was added to each well of the VetMIC plate. The plates were incubated at 37 °C for 48 h in anaerobiosis. The minimum inhibitory concentrations (MICs) were determined by spectrophotometric analysis of the plates at 620 nm and compared to the breakpoints proposed by EFSA for obligate heterofermentative *Lactobacillus* spp.[34].

To evaluate the hemolytic properties, *W. cibaria* SP7 and SP19 were anaerobically cultured on Columbia agar plates (Oxoid, Altrincham, England) containing 5% (w/v) defibrinated sheep blood (Biolife, Milano, Italy) at 37 °C for 48 h. *Staphylococcus aureus* ATCC 6538 was used as a positive control for hemolysis. The presence of β- or α-hemolysis was indicated by a clear or greenish zone around the colonies, respectively.

### Whole Genome Sequencing and Analysis

#### Genomic DNA Extraction and Sequencing

Genomic DNA was extracted from exponential phase MRS broth culture of *W. cibaria* SP7 and *W. cibaria* SP19 using the NucleoSpin Tissue kit according to the manufacturer’s instructions (Macherey–Nagel, Düren, Germany). Genomic DNA was sequenced at Fasteris (Geneve, Switzerland) using an Illumina MiSeq operating with V3 chemistry in 300X2 bp paired-reads. Basecalling was performed with MiSeq Control Software 2.4.1.3, RTA 1.18.54.0, and CASAVA-1.8.2.

#### Genome Assembly and Annotation

Raw Fastq reads obtained in Illumina were screened for quality by FastQC software (https://www.bioinformatics.bbsrc.ac.uk/projects/fastqc), retaining the reads with Phred score > 30. Genome assembly was performed using SPAdes (v3.11.1) [35], resulting in 64 contigs for *W. cibaria* SP7 and 25 contigs for *W. cibaria* SP19. Genome annotation was performed with Prokka (v1.13.3) [36]. Putative virulence factors were assessed by BLASTn with the VFDB [37] and MvirDB [38] databases. Putative antibiotic resistance genes were identified by BLASTn using ARDB database 1.1 [39], and by the Resistance Gene Identifier of the CARD database [40]. ResFinder 2.1 [41] was used to detect acquired antimicrobial resistance genes. Bacteriocin-coding genes were searched using BAGEL4 [42]. The presence of plasmids was assessed by PlasmidFinder 1.3 [43]. The presence of CRISPR-Cas systems was evaluated using the online tool CRISPRCasFinder [44].

To evaluate the pan- and core-genome of *W. cibaria*, all publicly available complete genomes (nine strains) and the two newly sequenced strains were analyzed using Roary (v3.12.0) [45]. The choice to consider only complete genome sequences was made since it has been previously evidenced that incomplete genomes highly affect the core-genome identification within a species [46]. The core gene alignment obtained with Roary was then used in RAxML (v8.2.12) to build a maximum-likelihood phylogenetic tree. The tree was visualized using iTOL [47]. Orthogroups (OGs) were inferred with OrthoFinder (v2.2.3) [48], and the gene content analysis was performed by mapping the OGs to the eggNOG bacterial database using eggNOG-mapper (v1.0.3) [49]. The gene content per OG for each strain (considering COG categories) was visualized with MeV [50].

### Statistical Analysis

Statistical analyses were performed using GraphPad Prism version 5 (GraphPad Software, San Diego, CA, USA). Phenotypic data were analyzed by two-way ANOVA with Bonferroni post-hoc test, with *P* < 0.05 considered significant.

## Results

### Phenotypic Characterization of *W. cibaria* SP7 and SP19

#### Resistance to Low pH and Bile Salts

**Fig. 1:**
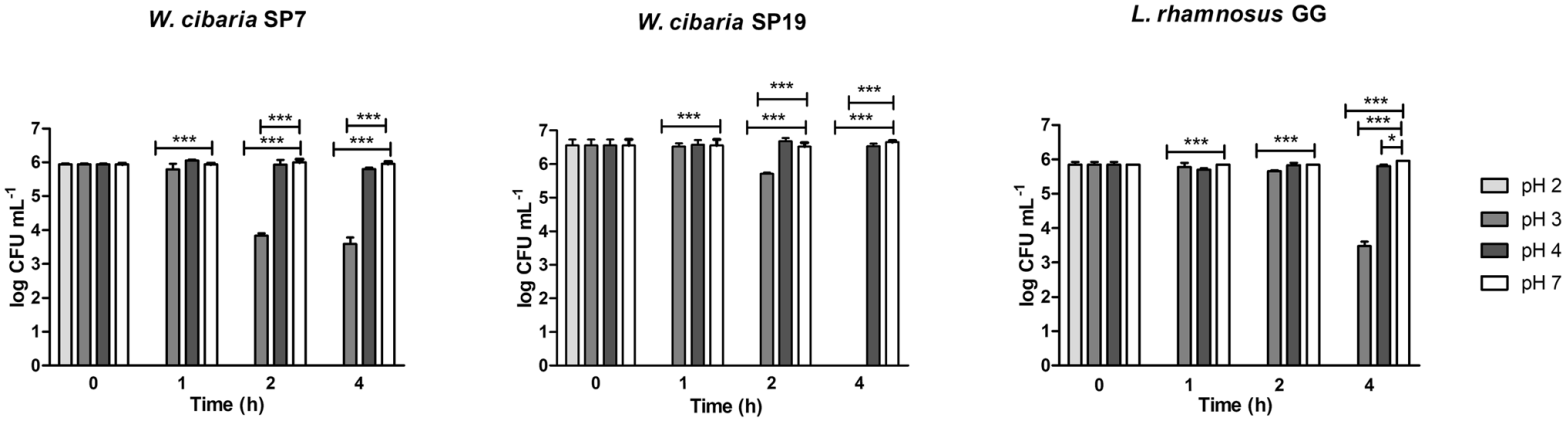
Survival of *W. cibaria* SP7 and SP19 at various pH as determined by viable bacteria counts. The probiotic *L. rhamnosus* GG was included as a control strain. Results are presented as mean±SD of three experiments; *P<0.05, ***P<0.001

The results of the acid tolerance test are shown in Fig. 1. No viable bacterial cells were detected after the first hour of exposure to pH 2, suggesting that all three strains were severely impaired upon acid shock. Exposure to pH 3 resulted in a decrease in viability for all strains; after 2 h, a decrease of 2 log and 1 log was detected for *W. cibaria* SP7 (*P* < 0.001) and SP19 (*P* < 0.01), respectively, as compared to pH 7. In contrast, for *L. rhamnosus* GG, significant growth reduction was observed only after 4 h (*P* < 0.001). At this time point, the concentration of viable *W. cibaria* SP19 cells declined to undetectable levels. All tested strains had a high survival rate when exposed to pH 4; only the viability of *L. rhamnosus* GG decreased significantly after 4 h incubation (*P* < 0.01) when compared to control.

**Fig. 2:**
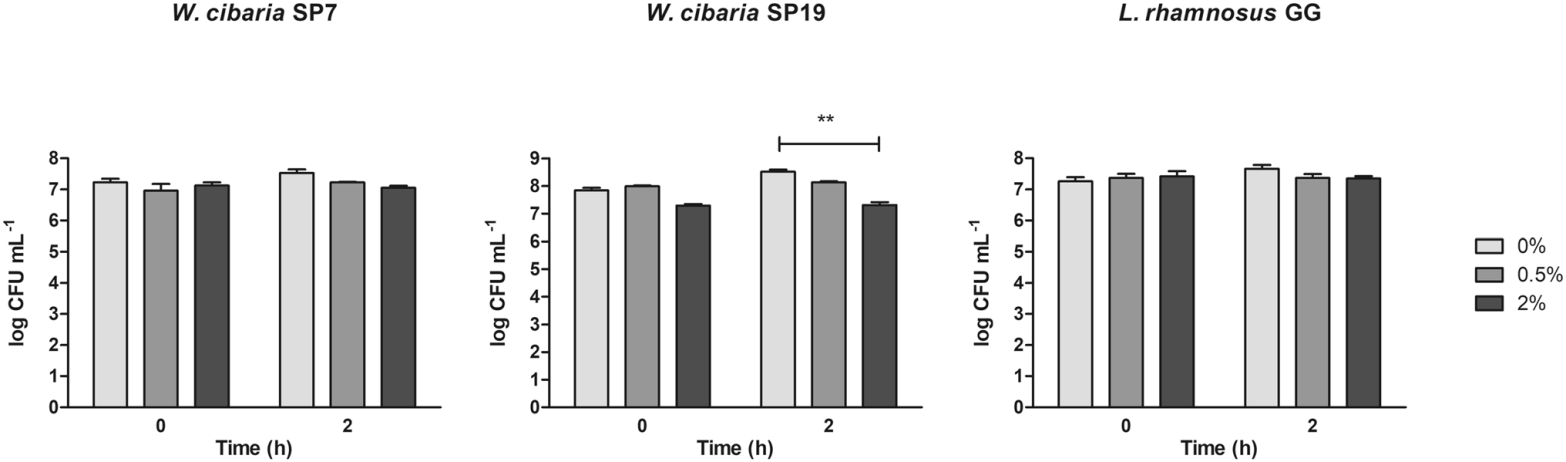
Survival of *W. cibaria* SP7 and SP19 at different bile salt concentrations as determined by viable bacteria counts. The probiotic *L. rhamnosus* GG was included as a control strain. Results are presented as mean ± SD of three experiments; **P < 0.01

The results of the bile tolerance test are shown in Fig. 2. All tested strains demonstrated good survival over the 2 h of exposure to concentrations of bile salts as high as 2%, with only a reduction in microbial counts as compared to 0% bile salts detected for SP19 (*P* < 0.01).

#### Aggregation and Co-Aggregation Tests

**Fig. 3:**
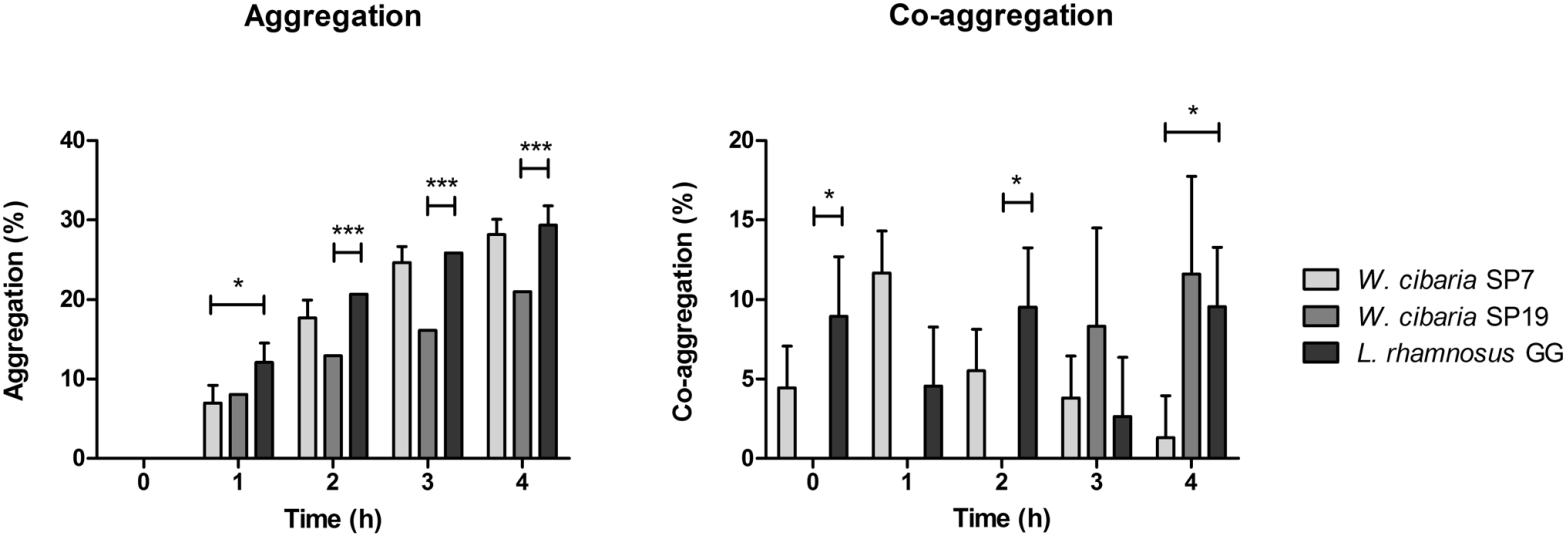
Aggregation and co-aggregation of *W. cibaria* SP7 and SP19 with *S. enterica* UC3605. The probiotic *L. rhamnosus* GG was included as a control strain. Results are presented as mean percentage ± SD of three experiments; *P < 0.05, ***P < 0.001

As shown in Fig. 3, the aggregation of *W. cibaria* SP7 was very similar to the aggregation of *L. rhamnosus* GG over the 4 h incubation, whereas *W. cibaria* SP19 exhibited significantly less aggregation. Neither the two *W. cibaria* isolates nor *L. rhamnosus* GG exhibited co-aggregative abilities with *E. coli* ATCC 25,922. Co-aggregation with *S. enterica* UC3605 was not observed for *W. cibaria* SP19 up to 2 h of incubation; afterwards, the percentage of co-aggregation was comparable to *L. rhamnosus* GG. In contrast, co-aggregation values for *W. cibaria* SP7 reached a maximum after 1 h of incubation and then progressively declined, becoming barely detectable after 4 h of incubation.

#### Adhesion to Cells and Porcine Gastric Mucin

**Fig. 4:**
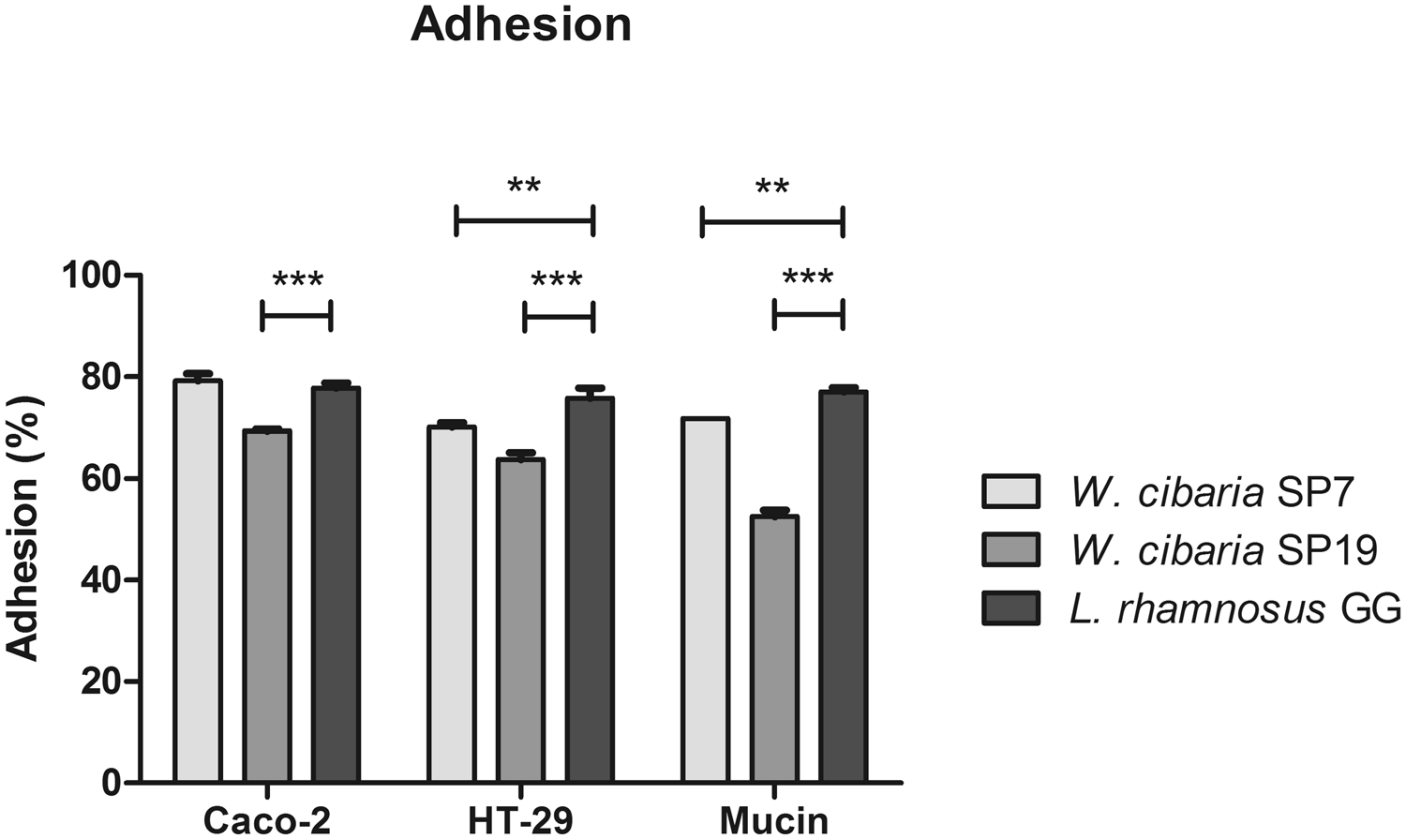
Adhesion of *W. cibaria* SP7 and SP19 to Caco-2, HT-29, and mucin. The probiotic *L. rhamnosus* GG was included as a control strain. Results are presented as mean percentage ± SD of two experiments; **P < 0.01, ***P < 0.001

All tested strains demonstrated a capacity to adhere to porcine gastric mucin at varying levels. *L. rhamnosus* GG exhibited the highest adhesion (77 ± 0.85%), followed by *W. cibaria* SP7 (72 ± 0.0%) and *W. cibaria* SP19 (53 ± 1.2%). Significant differences were observed between the adhesion of SP19 (*P* < 0.001) and SP7 (*P* < 0.01) compared to *L. rhamnosus* GG. *W. cibaria* SP7 and *L. rhamnosus* GG exhibited the same adhesion to Caco-2 cells (78 ± 1%), whereas *W. cibaria* SP19 exhibited less adhesion (69 ± 0.28%;i *P*< 0.001). In regards to HT-29 cells, *L. rhamnosus* GG displayed a percentage of adhesion (76 ± 2.0%) that was significantly higher than that of *W. cibaria* SP19 (64 ± 1.3%) and of *W. cibaria* SP7 (70 ± 0.8%) (Fig. 4). No significant differences were found between strains considering the percentage of adhesion to Caco-2 and HT-29 cells.

#### Antagonistic Activity Against Pathogens

The CFSs of both *W. cibaria* strains and *L. rhamnosus* GG exhibited antagonistic activity against reference *E. coli* and *S. enterica* strains. After 24 h of incubation with the *W. cibaria* SP7 supernatant, the growth of *E. coli* and *S. enterica* decreased 96 ± 1.3% (*P* < 0.001) and 94 ± 0.8% (*P* < 0.001), respectively. Similar percentages of inhibition were observed in *E. coli* (98 ± 1.2%; *P* < 0.001) and *S. enterica* (96 ± 0.0%; *P* < 0.001) when exposed to *W. cibaria* SP19 CFS. Analogously, *L. rhamnosus* GG significantly inhibited the growth of *E. coli* and *S. enterica* (97 ± 1.2% and 95 ± 0.8%, respectively; *P* < 0.001). Neutralization of CFS resulted in complete reversal of the inhibitory effects (data not shown), suggesting a major role of organic acids in supporting the antagonistic activity of the three tested bacteria against pathogens.

#### Carbohydrate Fermentation Patterns

The results of the carbohydrate fermentation test are shown in Table 1. Both *W. cibaria* SP19 and SP7 were able to ferment a number of sugars, including L-arabinose, D-ribose, D-glucose, D-fructose, D-mannose, N-acetylglucosamine, D-maltose, and potassium gluconate. In addition, *W. cibaria* SP19 was able to ferment D-xylose, D-galactose, and D-saccharose, as well as β-glucosides D-cellobiose, amygdalin, arbutin, esculin, gentobiose, and salicin.

**Table 1:** Carbohydrate fermentation patterns of *W. cibaria* SP7 and SP19 strains

Sugar	*W. cibaria* SP7	*W. cibaria* SP19
L-Arabinose	+	+
D-Arabinose	-	-
D-Ribose	+	+
D-Xylose	-	+
L-Xylose	-	-
D-Galactose	-	+
Methyl-d-xylopyranoside	-	-
D-Glucose	+	+
D-Fructose	+	+
D-Mannose	+	+
L-Sorbose	-	-
L-Rhamnose	-	-
Methyl-d-mannopyraranoside	-	-
Methyl-d-glucopyranoside	-	-
N-Acetylglucosamine	+	+
Amygdalin	-	+
Arbutin	-	+
Esculin	-	+
Salicin	-	+
D-Cellobiose	-	+
D-Maltose	+	+
D-Lactose	-	-
D-Melibiose	-	-
D-Trehalose	-	-
Gentiobiose	-	+
Potassium gluconate	+	+
D-Saccharose	-	+
D-Raffinose	-	-
D-Fucose	-	-
L-Fucose	-	-

The production of EPS was assessed by evaluating the color of colonies grown on mod-MRS agar plates containing various carbohydrates. *W. cibaria* SP19 resulted in slimy colonies, indicating EPS synthesis and secretion only in the presence of sucrose, whereas *W. cibaria* SP7 grew as red colonies on the same plates, showing no ability to produce EPS (data not shown).

#### Safety Assessment

The MICs of the tested antibiotics are listed in Table 2. For both *W. cibaria* strains, the MICs did not exceed the MIC cut-off values proposed by EFSA for obligate heterofermentative *Lactobacillus* spp.

**Table 2:** Minimal inhibitory concentrations of tested antibiotics towards *W. cibaria* SP7 and SP9

**Antibiotic**	**MIC (mg L**^**−1**^)		**EFSA cut-off values (mg L**^**−1**^)
	***W. cibaria*** SP7	***W. cibaria*** SP19	***Lactobacillus*** obligate heterofermentative
Ampicillin	0.5	0.5	2
Gentamicin	< 0.5	1	16
Kanamycin	8	16	32
Streptomycin	8	8	64
Neomycin	1	1	-
Tetracycline	8	8	8
Erythromycin	0.5	0.25	1
Clindamycin	0.12	0.06	1
Chloramphenicol	4	4	4

Neither β-hemolytic nor α-hemolytic phenotypes were detected for *W. cibaria* SP7 and SP19 grown on blood agar plates. In contrast, the positive control *S. aureus* ATCC 6538 exhibited the expected β-hemolysin activity (data not shown).

### Features of the *W. Cibaria* SP7 and SP19 Genomes

#### Genes Associated with Persistence in the Host and Probiotic Function

The general genomic properties of *W. cibaria* SP7 and *W. cibaria* SP19 are presented in Table 3 and Fig. S1. In the preliminary RAST annotation, both the SP7 and SP19 genomes contained a number of genes encoding enzymes required for the metabolism of mannose, D-ribose, xylose, D-gluconate and ketogluconates, and L-arabinose. The SP19 genome also included genes encoding enzymes required for the utilization of fructose, D-galacturonate, and D-glucuronate. Liver-produced β-D-glucuronides are detoxification products excreted into the mammalian gut, and several symbiotic microorganisms have adapted to exploit host-derived glucuronides as a source of carbon for growth. Both strains harbored putative PTS transporter and permease genes for sucrose, lactose, trehalose, and galactose utilization. Genome mining with Prokka annotation identified genes involved in chitin and N-acetylglucosamine utilization, coding for N-acetylglucosamine-6-phosphate deacetylase (NagA), N-acetylglucosamine-6-phosphate deaminase (NagB), and N-acetylglucosamine repressor (NagC). Notably, two beta-glucoside operons were found in both genomes, the *bglABFHK* operon and *licABCT* operon (with SP7 lacking *bglB* and *licBC* in the two operons, respectively), which are responsible for the catabolism of polysaccharides present in the plant cell wall, such as cellobiose, salicin, and arbutin. *W. cibaria* SP19 possessed putative enzymes required for glycogen synthesis in bacteria, including the glycogen synthase (GlgA), 1,4-alpha-glucan branching enzyme (GlgB), glucose-1-phosphate adenylyltransferase (GlgC), glycogen biosynthesis protein glucose-1-phosphate adenylyltransferase (GlgD), and glycogen phosphorylase (GlgP), responsible for glycogen and starch breakdown.

**Table 3:** General genomic properties of *W. cibaria* SP7 and *W. cibaria* SP19

Features	*W. cibaria* SP7	*W. cibaria* SP19
Genome size (bp)	2,007,138	2,499,540
GC content (%)	44.3	44.8
Total CDS	2088	2354
rRNA	4	2
tRNA	49	53
5S rRNA	3	1
CRISPR	0	1
Coverage	346x	310x

We also found several genes associated with stress resistance in SP7 and SP19, including genes encoding for resistance to osmotic stress (aquaporin Z, glycerol uptake facilitator protein, and OpuABC and GbuA gene clusters related to the uptake and biosynthesis of osmo-protectants choline and betaine). Bile stress has been shown to induce oxidative stress, and a number of genes involved in resistance to oxidative stress were observed in the two *Weissella* isolates. These genes include proteins with ferroxidase activity, redox-sensitive transcriptional regulator (AT-rich DNA-binding protein), and genes involved in the biosynthesis and reduction of the cellular antioxidant glutathione. Both strains harbored *hflX* and *hflK*, which are associated with the 50S ribosomal subunit and may play a role during protein synthesis or ribosome biogenesis. Finally, genes that confer resistance to cold (CspLA and CspB proteins) and 11 genes conferring resistance to heat shock were found in both genomes. They belong to the heat shock *dnaK* gene cluster extended subsystem and include the DnaK (HrcA-GrpE-DnaK-DnaJ) operon. Other genes are involved in protecting ribosomal function during heat shock and other stresses *(lepA, prmA, rdgB, smpAB, rsmABCDEFGHI).* No bile salt hydrolase (*bsh*) genes were present.

Gene prediction with Prokka identified genes specifically associated with adhesion. Specifically, the presence of genes coding for capsular proteins (*cps12A* and *cap8A*) and fibronectin-binding protein (*fbpA*) was demonstrated, as well as three and two copies of mucin-binding protein (*mucBP*) in SP7 and SP19, respectively. Moreover, SP7 and SP19 harbored two and three copies of the LPXTG specific sortase gene, respectively, along with three copies of the gene coding for the biofilm regulatory protein A precursor (*brpA*). EPS clusters, namely EpsDFHJLE, were identified in the two strains, with both lacking EpsA. EPS may contribute to cell adhesion and exert a protective function in the bacterial cells against the environment. Notably, both strains harbored genes encoding enzymes predicted to be involved in N-acetylglucosamine catabolism and synthesis (*nagA, nagB, nagC, glmS, glmM, glmU, manX*). However, the SP19 genome also harbored genes involved in dTDP-rhamnose biosynthesis (*rfbC*, *rmlD*), whereas SP7 does not harbor these genes. Moreover, multiple copies of glycosyltransferase genes were found in both strains (*gtf1*, *gtfC*), as well as maltose metabolism genes (*maa*, *malP*, *malL*).

Gene prediction by Prokka highlighted the presence of a putative gene coding for the bacteriocin Colicin V, in both strains of *W. cibaria*. However, a more in-depth characterization with BAGEL4 did not detect a related gene cluster for the bacteriocin production.

CRISPRCasFinder identified one CRISPR-Cas system in *W. cibaria* SP19, including CAS-TypeIIA proteins (Cas1, Cas2, Cas9, Csn2).

A summary of the main genes identified in the two newly sequenced strains of *W. cibaria*, that can be potentially associated to probiotic functions, are reported in Table 4.

**Table 4:** List of genes putatively related to probiotic function identified in the genomes of *W. cibaria* SP7 and *W. cibaria* SP19

General function	Gene	Predicted function
adhesion	*cps12A*	capsular polysaccharide phosphotransferase
	*cap8A*	capsular polysaccharide type 8 biosynthesis protein
	*fbpA*	fibronectin-binding protein
	*mucBP*	mucus-binding protein
	*epsDFHJLE*	exopolysaccharide synthesis
	*srt*	sortase
	*brpA*	biofilm regulatory protein
stress resistance	*dnaK*	chaperone protein
	*hflX, hflK*	GTPase, modulator of FtsH protease
	*aqp Z*	aquaporin
	*gla*	glycerol facilitator-aquaporin
	*opuABC*	glycine betaine transport system permease
	*gbuA*	glycine betaine/carnitine transport ATP-binding protein
	*gshAB*	glutathione biosynthesis bifunctional protein
sugar metabolism	*nagA, nagB, nagC*	N-acetylglucosamine-6-phosphate deacetylase, glucosamine-6-phosphate deaminase, N-acetylglucosamine repressor
	*glmM, glmU, glmS*	phosphoglucosamine mutase, glucosamine bifunctional protein, glutamine–fructose-6-phosphate aminotransferase
	*manX*	PTS system mannose-specific
	*gtf1, gtfC*	glycosyltransferase
	*rfbC, rmlD *^*1*^	dTDP-4-dehydrorhamnose 3,5-epimerase, dTDP-4-dehydrorhamnose reductase
	*maa, malP, malL*	maltose O-acetyltransferase, maltose phosphorylase, oligo-1,6-glucosidase
	*glgA, glgB, glgC, glgD, glgP *^*1*^	glycogen synthase, 1,4-alpha-glucan branching enzyme, glucose-1-phosphate adenylyltransferase, glycogen biosynthesis protein, glycogen phosphorylase
	*bglABFHK *^*2*^	beta-glucosidase
	*licABCT *^*2*^	PTS system lichenan-specific

^1^ Genes present only in *W. cibaria* SP19

^2^ *bglB* and *licBC* lacking in *W. cibaria* SP7

#### Genes Associated with Safety

Analysis of the SP7 and SP9 genomes by comparisons to the VFDB and MvirDB databases indicated that no putative virulence-associated genes were present. The Antibiotic Resistance Database (ARDB) identified no gene above the cut-off (70% identity, *P* < 0.0001). Analysis by ResFinder (selected % ID threshold: 98.00%, selected minimum length: 60%) identified no acquired antimicrobial resistance genes in the genome of either strain. Finally, interrogation of the comprehensive Antibiotic Resistance Database (CARD) resulted in no positive hits when selecting perfect and strict hits, or hits below a percentage identity of 45% when allowing loose hits. No plasmids were found by PlasmidFinder.

Two genes coding for hemolysin A (*TlyA*) and hemolysin-III-related protein were found in both SP7 and SP19.

#### Pan- and Core-Genome Analysis

Comparative genomics of the newly sequenced strains with complete *W. cibaria* genomes revealed the presence of 1323 core genes (99% ≤ strains ≤ 100%), 1360 shell genes (15% ≤ strains < 95%), and 2008 cloud genes (0% ≤ strains < 15%), resulting in a pan-genome of 4691 genes (Fig. S2a). Variations in the content of unique and new genes in the *W. cibaria* pan-genomes, as a function of the number of analyzed strains, are shown in Fig. S2b. The phylogenetic tree based on the core gene alignment of the 11 strains was re-rooted on strain SP7, serving as an outgroup (Fig. S2c). Interestingly, the two newly sequenced strains clustered differently, even if the isolation source was the same. Excluding the outgroup (SP7 strain), two main clades were highlighted: one including strains M2, CBA3612, and CH2, and the other including BM2, SRCM103448, SP19, CMS2, CMS3, and the proposed oral care probiotic strains CMS1 and CMU [51–53] (Fig. S2b).

Evaluating the pan-genome, 97% of the genes identified in all genomes were clustered into 2509 OGs, and 1724 were defined as core OGs (i.e., containing all species). OGs were then mapped to the bacterial eggNOG database to evaluate the main functional COG categories related to the pan-genome considering each strain (Fig. S3). The major fraction of OGs (24%) was grouped under the “S” category of “unknown function” (not included in Fig. S3). From the hierarchical clustering based on the Euclidean distance, two main clusters were shown, grouping the more and less abundant COG categories, respectively. Similarly, two main clades were formed by separating the two strains: CBA3612 and CH2. Considering the relative abundance profiles, no differences were highlighted between the strains when considering the most abundant COG categories, which were related to amino acid and carbohydrate transport and metabolism, and DNA replication and translation activities (E, G, J, L). However, some differences were found within the categories of “Cell envelope biogenesis, outer membrane” (M), “Transcription” (K), “Inorganic ion transport and metabolism” (P), and “Defense mechanisms” (V) (Fig. S3).

## Discussion

Probiotics are gaining interest from research and the food industry as a possible strategy for controlling intestinal infections in livestock and to reduce antibiotic use in animal feed, thus helping contain the risks associated with the spread of antibiotic-resistance genes or the contamination of animal-derived products with antibiotic residues. In addition, the inclusion of probiotics in cattle diets has the potential to improve gut function and increase the utilization of feed, ultimately enhancing the health and productivity of ruminant animals. In this study, the potential of two *W. cibaria* strains newly isolated from cattle in Kuwait as animal probiotics was investigated based on their whole genome sequences and corresponding phenotypes. The phenotypic assessment of *W. cibaria* SP7 and SP19 showed that both strains possess good tolerance to low pH and bile salts in vitro to varying degrees. The comparison with well-known probiotic *L. rhamnosus* GG indicated similar levels of pH and bile resistance. Although data are available only for a limited number of strains, resistance of *W. cibaria* strains to a pH as low as 2.5 and 0.3% bile salts has been described in some previous studies on fermented food and intestinal isolates of human and animal origin [6, 54–57]. The bile salt resistance of *Weissella* spp. is likely to be considered a strain-dependent property similar to what has been observed in *Lactobacillus* and *Bifidobacterium* spp.[58]. A *W. cibaria* WD2 isolate obtained from fermenting cassava mash survived in simulated gastric and intestinal transit, tolerating acid (pH 2) and bile salt (1%) [59], whereas *W. cibaria* KCTC 3746 isolated from kimchi was not able to retain its viability when exposed to 0.3% bile salts in the presence of pancreatin [31]. At the genomic level, both strains under investigation seem to carry a good set of genes to counteract stress; some of them are frequently found in lactic acid bacteria, such as the chaperone system DnaK, which has been demonstrated to play a role in the acid resistance response [60, 61]. In contrast, little is known about the HflX GTPase, but proteomic studies in probiotic *Lactobacillus gasseri* ATCC 33323 suggest that HflX may act as a key regulator during stress [62]. Furthermore, it must be stressed that the occurrence of a coding gene not always implies the formation of a functional gene product or a direct involvement in a specific biological activity. We found no evidence of bile salt hydrolases in the *W. cibaria* strains under study. In addition to bile acid modification activity, bile tolerance develops through multiple mechanisms that can be bile-specific, such as efflux pumps and cell wall and membrane modification, as well as mechanisms underlying the overall bacterial capacity to maintain intracellular homeostasis [58]. Notably, recent studies relying on omics technologies have allowed the detection of numerous proteins for which their expression is influenced by bile salt stress, including proteins involved in carbohydrate metabolism, cell envelope and lipid metabolism, efflux pumps, and proteins related to the general stress response [63].

Phenotypic test results indicated the ability of both tested strains to ferment carbohydrates such as L-arabinose, D-ribose, D-glucose, D-fructose, D-mannose, N-acetylglucosamine, D-maltose; D-saccharose, D-galactose, and gentiobiose were fermented only by *W. cibaria* SP19. More than 12% and 15% of the identified genes in the two bovine isolates, *W. cibaria* SP7 and SP19, respectively, were involved in carbohydrate metabolism. In this context, Lynch et al. [64] reported that the sourdough isolate *W. cibaria* MG1 has the potential to metabolize galactose, maltose, fructose, ribose, xylose, sucrose, and gluconate. They also identified putative phosphotransferase systems (PTSs) for N-acetyl-D-glucosamine, cellobiose, mannose, b-glucosides, and fructose. In contrast to *W. cibaria* SP7, *W. cibaria* SP19 exhibited an ability to utilize sugars widely distributed in plants, namely D-xylose and β-glucosides, including arbutin, esculin, and salicin, suggesting some level of adaptation to the plant-based diet of ruminants.

Interestingly, *W. cibaria* strain SP19 also possessed putative enzymes conferring an ability to synthesize glycogen and a glycogen phosphorylase involved in glycogen catabolism*.* The glycogen metabolic pathway has been detected in probiotic strains or intestinal isolates belonging to the *Lactobacillus* genus, suggesting potential involvement of this pathway in the persistence and probiotic functionalities of lactobacilli in the gut [65, 66].

At the phenotypic level, both *W. cibaria* strains exhibited adhesion properties to epithelial cell lines and mucin in vitro*,* though significantly lower percentages were detected compared to *L. rhamnosus* GG. These results are consistent with previous findings [6, 55] indicating the in vitro adhesion capacity of *W. cibaria* strains to Caco-2 cells. *W. cibaria* KCTC 3746 exhibited a high percentage of auto-aggregation after 24 h incubation and effectively adhered to human intestinal epithelial HT-29 cells [31]. At the genomic level, the two isolates harbored genes encoding proteins specifically associated with adhesion, such as fibronectin-binding protein [67] and mucin-binding protein. No mucus-binding proteins were detected in *W. cibaria* KACC 11,862 in a previous study [68]. The presence of sortases, enzymes anchoring surface proteins to the cell wall of Gram-positive bacteria, could also play an important role. Deletion of the sortase-encoding gene reduced adherence of *Ligilactobacillus* (formerly *Lactobacillus*) *salivarius* UCC118 to epithelial cells [69].

Antagonistic activity and the production of antimicrobial compounds is another very important characteristic needed to inhibit the growth of pathogenic bacteria. The ability of *W. cibaria* SP19 and SP7 to exert effective antimicrobial activity against *E. coli* and *Salmonella* spp., which are major causes of calf enteric disorders in Kuwait dairy herds [70], was associated with the production of organic acids and the resulting decrease in pH. *W. cibaria* is able to produce several antimicrobial substances; recently, the antibacterial activity of the oral probiotic *W. cibaria* CMU was linked to its ability to produce H_2_O_2_, organic acids, oleic acid, and specific proteins, such as N-acetylmuramidase [53]. The production of bacteriocins was described previously in a limited number of *Weissella* strains belonging to the species *W. cibaria*, *W. paramesenteroides,* and *Weissella hellenica* [71–75]. Unfortunately, we were not able to highlight any production of antimicrobial substances other than organic acids in the growth medium or putative bacteriocin gene clusters in the genomes of the strains under investigation.

However, the production of antimicrobial compounds is only one of several mechanisms through which probiotics can prevent or reduce the colonization and infection of livestock and humans. In our experimental conditions, co-aggregation of *W. cibaria* SP19 with the gut pathogen *S. enterica*, though expressed at a low level, was comparable to that of *L. rhamnosus* GG after 4 h incubation, and the ability of probiotic strains to co-aggregate with pathogens is thought to play an important role in their protective function [76].

In regards to safety, we exploited whole genome sequencing data analysis in combination with in vitro testing to conduct a comprehensive assessment of the antibiotic resistance and pathogenic potential of *W. cibaria* isolates, as recommended by Hill et al. [77]. We found no evidence of phenotypic antibiotic resistance characteristics in the two *W. cibaria* strains we investigated. Few studies are available on the antibiotic resistance profiles of *Weissella* species and, unlike other lactic acid bacteria genera, no specific MIC breakpoint values have been defined by EFSA for the assessment of antimicrobial susceptibility. Jeong and Lee [78] found that the *W. cibaria*, *W. confusa,* and *W. paramesenteroides* strains are resistant to streptomycin, and *W. cibaria* strains to penicillin G. Low rates of resistance to aminoglycosides have been reported in the *Leuconostoc/Weissella* group [79, 80], and *Leuconostoc* strains of food origin have been susceptible to beta-lactams in other studies [80–82]. In regards to the resistance to penicillin G, Jeong and Lee [78] reported that the results from the disk diffusion test were inconsistent with those from the microdilution broth assay, indicating the need to define specific cut-off values for *Weissella* spp. However, in our study, the search for antibiotic resistance determinants identified no specific gene in either strains, confirming the antimicrobial susceptibility testing results.

In line with previous findings [68], both *W. cibaria* strains harbored genes for hemolysin or hemolysin-like proteins, but the presence of such genes was not associated with the expression of a hemolytic phenotype, further confirming the safety profiles of the isolates.

Comparative genomic analysis showed that the *W. cibaria* pan-genome is still “open”, as nearly 25 new genes are included for each additional genome considered. This is also due to the low number of genomes currently available for *W. cibaria*. However, the presence of variable genetic content that relies on strain-specific genes (unique) is suggested.

Although a lot of commercial probiotic preparations are currently available for a wide range of conditions, it is clear that not all organisms are well suited for all applications. Notably, species-specificity [83, 84] and intestinal origin [85, 86] of bacterial strains have been reported as relevant factors influencing probiotics efficacy.

The results of this study add to our understanding of the genomics and metabolic capabilities of gut-associated *W. cibaria* species, and shed light on potential mechanisms of host-microbe interaction that represent a crucial input for the probiotics development process. Since functional attributes of specific strains can be related to their evolution and the mutualistic relationship established with different hosts [87]*,* our findings could contribute to select probiotics which are more likely to colonize and maintain in the intestine of cow, and to exert specific physiological effects on the host. Future investigations are necessary to ascertain whether the in vitro characteristics of our *W. cibaria* strains may reflect the in vivo behavior of the isolates when administered to control infection and reduce the use of antibiotics under field conditions.

In conclusion, the integration of whole-genome sequencing and phenotyping provides insight into the genomics and physiology of *W. cibaria* strains of animal origin and demonstrated that specific strains belonging to this species have relevant features that make them suitable for probiotic applications. To the best of our knowledge, this study is the first to report the draft genome sequences of *W. cibaria* strains derived from bovine intestine. Our results indicate that *W. cibaria* strain SP19, and to a lesser extent strain SP7, possesses several in vitro probiotic capacities, such as good tolerance to acidic pH and bile salts, adherence to intestinal cells, and antagonistic activity against pathogens. These properties, together with genetic characteristics, such as the lack of transferable antibiotic resistance determinants and pathogenicity factors, and the presence of genes revealing adaptation to the gut environment, suggest a potential use of these strains as novel probiotics tailored to prevent intestinal infections in cattle production. In vivo trials are mandatory to confirm the in vitro results of this study.

## Data Availability

The whole genome sequencing projects for *W. cibaria* strains SP7 and SP19 have been deposited at DDBJ/ENA/GenBank under accession numbers SDGJ00000000 and SDGK00000000, and in the NCBI Sequence Read Archive (SRA) database with accession numbers SRR8486238 and SRR8494494
